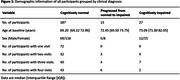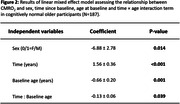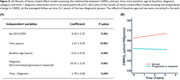# Longitudinal changes in cerebral metabolic rate of oxygen in older adults without and with cognitive impairment

**DOI:** 10.1002/alz.093651

**Published:** 2025-01-09

**Authors:** Jiani Wu, Kumiko Oishi, Anja Soldan, Corinne Pettigrew, Zixuan Lin, Yuxin Zhu, Dengrong Jiang, Xin Li, Abhay Moghekar, Peiying Liu, Kenichi Oishi, Marilyn S. Albert, Hanzhang Lu

**Affiliations:** ^1^ Johns Hopkins University School of Medicine, Baltimore, MD USA

## Abstract

**Background:**

Cerebral metabolic rate of oxygen (CMRO2) denotes the amount of O2 that the brain consumes. Changes in CMRO2 during aging and neurodegeneration have not been fully characterized. Using a non‐invasive, non‐contrast MRI CMRO2 technique, the present study reports CMRO2 changes in older adults from a total of 526 measurements, the largest CMRO2 dataset to date.

**Method:**

Experimental procedure: Analyses included 227 participants from the Biomarkers‐for‐Older‐Controls‐at‐Risk‐for‐Dementia (BIOCARD) cohort. Figure 1 shows the demographic information. Participants were categorized into 3 diagnostic categories: cognitively normal individuals who remained cognitively normal (n=187), individuals who progressed from normal cognition to Mild Cognitive Impairment (MCI) or dementia during follow‐up (n=13), and individuals with MCI/dementia at baseline (n=27). Participants were scanned on a 3T MRI (Philips) in a longitudinal study design. On average, participants had 2 scans (range = 1‐4) collected over 3.1 years of follow‐up. CMRO2 was estimated from the arterio‐venous difference in oxygen content, using a non‐invasive MRI technique. Data analysis: Linear mixed effect models were used to examine longitudinal changes in CMRO2 over time, as well as the effect of impairment (MCI/dementia) on CMRO2.

**Result:**

In individuals who remained cognitively normal during follow‐up (Figure 2), there was an increase in CMRO2 over time (p<0.001), suggesting that the brain is “working harder” to compensate for its lower efficiency. However, the rate of increase in CMRO2 was attenuated among individuals with higher baseline ages (p=0.039). There were also main effects of baseline age (p=0.001) and sex (p=0.014), indicating lower CMRO2 levels among older than younger adults and among men than women. Next, we included participants who were cognitively impaired and those who progressed from normal cognition to cognitive impairment (Figure 3A). CMRO2 was lower among progressors and impaired participants than those who remained normal (p=0.001). There was an interaction effect between time and diagnosis category (p=0.044), indicating that participants who were impaired at baseline or progressed to impairment showed a different pattern of CMRO2 change over time compared to those who remained normal (Figure 3B).

**Conclusion:**

CMRO2 revealed a non‐monotonic change in aging and cognitive impairment, suggesting a multi‐factorial and/or multi‐phase alteration of brain metabolism.